# PEZO-1 is not required for AMsh glial responses to mechanical stimulation and does not play a major role in nose touch avoidance in *C. elegans*

**DOI:** 10.17912/micropub.biology.001668

**Published:** 2025-06-19

**Authors:** Jesus Fernandez-Abascal, Jesse D. Hall, Laura Bianchi

**Affiliations:** 1 Department of Physiology and Biophysics, University of Miami Health System, Miami, Florida, United States; 2 Andalusian Centre for Developmental Biology (CABD), CSIC-Universidad Pablo de Olavide-Junta de Andalucía; 3 Department of Molecular Biology and Biochemical Engineering, Universidad Pablo de Olavide

## Abstract

In *Caenorhabditis elegans* , nose touch avoidance behavior is regulated by the Amphid Sheath (AMsh) glial cells via release of GABA. AMsh glia themselves exhibit calcium responses to mechanical stimulation, although the mechanosensitive channel responsible remains unidentified . Here, we investigated whether *pezo-1* , the sole *C. elegans* homolog of mammalian PIEZO1 and PIEZO2, mediates AMsh glial mechanosensitivity and contributes to nose touch avoidance. We examined behavioral responses in three *pezo-1* mutant strains, including a full gene deletion and two truncation alleles lacking the pore-forming transmembrane domains. In addition, we monitored calcium transients in the full gene deletion strain. While *pezo-1 ( av149 )* mutants showed a slight reduction in nose touch response, *pezo-1 ( av240 )* and *pezo-1 ( sy1199 )* mutants behaved like wild-type animals. In vivo calcium imaging revealed that AMsh glial responses to touch were preserved in *pezo-1 ( av240 )* mutants, with no significant difference in peak calcium signals compared to wild-type. These findings indicate that *pezo-1* is not required for AMsh glial mechanosensory responses or nose touch avoidance behavior. Further research is needed to identify the channels and pathways mediating mechanotransduction in AMsh glia.

**Figure 1. Loss of PEZO-1 function does not affect AMsh glial calcium responses to mechanical stimulation or nose touch avoidance behavior.**
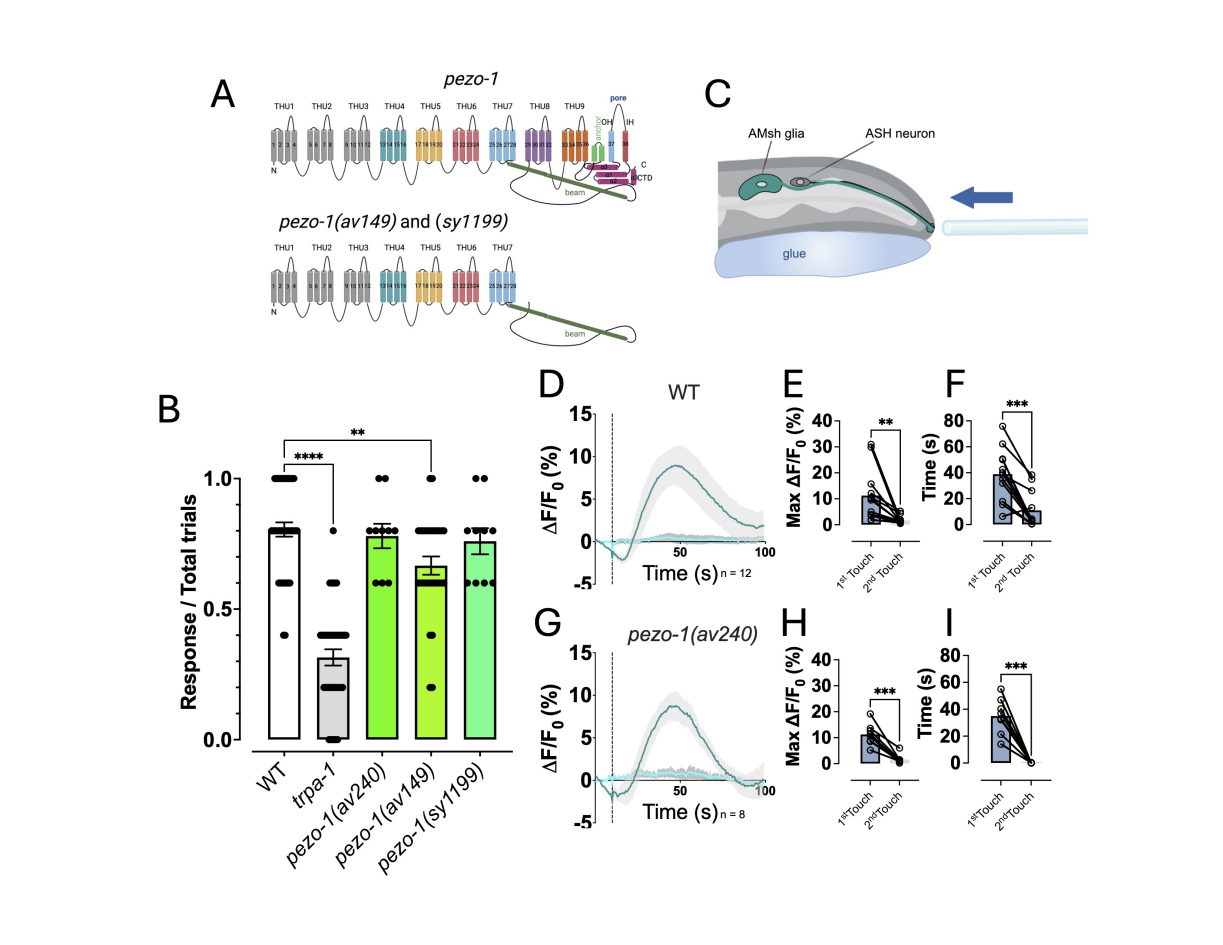
**(A)** Schematic representation of the predicted topology of PEZO-1 , a mechanosensitive ion channel composed of 38 transmembrane domains arranged into 9 transmembrane helix units (THUs). The channel also includes a central pore domain—comprising inner (IH) and outer ( OH ) helices—a transmembrane anchor domain, an intracellular beam domain, and a C-terminal domain (CTD) containing four intracellular α-helices. The anchor domain links peripheral mechanosensitive blades to the central pore, potentially influencing ion conduction and structural stability. The beam domain serves as a mechanical lever transmitting force to the pore, thereby regulating channel gating. In the *pezo-1 ( av149 )* (deletion) and *pezo-1 ( sy1199 )* (premature stop codon) alleles, transmembrane domains 29–38, including the pore region, are absent. **(B)** Nose touch avoidance behavior in wild-type, *trpa-1* (negative control), *pezo-1 ( av149 )* , *pezo-1 ( av240 )* , and *pezo-1 ( sy1199 )* animals. N = 40, 40, 10, 30, and 10, respectively. **(C)** Diagram of the *C. elegans* head depicting one of the two AMsh glial cells (green) and associated ASH sensory neurons. GCaMP-6s, a genetically encoded calcium indicator, is expressed in AMsh glia. Mechanical stimuli are applied to the nose tip using a glass probe controlled by a digital motion controller. **(D)** Calcium transients in AMsh glia in response to two consecutive nose tip stimulations in wild-type animals, measured as the change in GCaMP-6s fluorescence over time. The dashed line represents the time the touch stimulation was delivered. **(E)** Quantification of peak fluorescence changes following the first and second touch stimuli in wild-type animals. **(F)** Calcium transients decay time. (N = 12) **(G-I)** Calcium transients and corresponding quantification for *pezo-1 ( av240 )* mutants (N = 8), showing similar responses to wild type. Data are presented as individual data points with mean ± SEM in (B), mean ± SEM in (D) and (G), as individual data points with mean in (E), (F), (H), and (I). Statistics were by Anova with Tukey correction in B, and paired student's t-Test in E, F, H and I **, ***, and **** indicate p<0.01, 0.001, and 0.0001, respectively.

## Description

*C. elegans* responds to mechanical stimulation of the nose by initiating backward locomotion. This behavior is primarily mediated by a pair of head sensory neurons known as ASH (Kaplan & Horvitz, 1993). Upon nose touch, ASH neurons exhibit an increase in intracellular calcium that depends on the function of the putative mechanosensitive DEG/ENaC channel DEG-1 (Fernandez-Abascal et al., 2022; Geffeney et al., 2011; Kindt et al., 2007). This calcium influx triggers the release of glutamate and the neuropeptide NLP-3 , which activate downstream interneurons to initiate the escape response (Fernandez-Abascal et al., 2022; Hart et al., 1995). Recent studies have shown that ASH neuron activity is regulated by the associated Amphid Sheath (AMsh) glial cells through GABA signaling (Cheng et al., 2024; Duan et al., 2020; Fernandez-Abascal et al., 2022; Graziano et al., 2024). Specifically, GABA released from AMsh glia dampens ASH responses during repeated stimulation, preventing sensory overload and preserving touch sensitivity. Interestingly, AMsh glia themselves respond to mechanical stimulation with increases in intracellular calcium (Chen et al., 2022; Duan et al., 2020; Fernandez-Abascal et al., 2022). However, the identity of the mechanosensitive channel responsible for this glial response remains unknown.

In this study, we tested the hypothesis that the mechanosensitive ion channel *pezo-1* contributes to the Amphid Sheath (AMsh) glial response to mechanical stimulation and plays a role in the nose touch avoidance behavior of *C. elegans* . *pezo-1* is the sole *C. elegans* homolog of the mammalian PIEZO1 and PIEZO2 channels, which belong to a conserved family of mechanically-gated ion channels involved in diverse physiological processes (Coste et al., 2012). PIEZO1 is predominantly expressed in non-sensory tissues and is essential for vascular development, blood flow sensing, and red blood cell volume regulation (Cahalan et al., 2015; Li et al., 2014; Ranade et al., 2014; Zarychanski et al., 2012). It also contributes to bladder stretch sensing, cell migration, and epithelial cell extrusion (Gudipaty et al., 2017; Miyamoto et al., 2014), and has been implicated in neuronal development and cancer metastasis (McHugh et al., 2012; Pathak et al., 2014). In contrast, PIEZO2 is primarily expressed in sensory cells, including dorsal root ganglion (DRG) neurons and Merkel cells, where it mediates light touch sensation (Coste et al., 2012; Maksimovic et al., 2014; Woo et al., 2014). Genetic studies in mice and humans have shown that PIEZO2 is essential for proprioception, respiratory mechanosensation, and stretch sensing in internal organs such as the bladder (Chesler et al., 2016; Marshall et al., 2020; Nonomura et al., 2017; Szczot et al., 2018). Given these roles, we investigated whether *pezo-1* similarly contributes to mechanosensory function in AMsh glial cells and behavior in *C. elegans* . So far *C. elegans pezo-1* has been involved in food sensation, pharyngeal pumping, mating behavior, and crawling (Brugman et al., 2022; Hughes et al., 2022; Komandur et al., 2023; Millet et al., 2022; Short, 2022).

To investigate the role of *pezo-1* in AMsh glial calcium responses to mechanical stimulation and in nose touch avoidance behavior, we analyzed three different *pezo-1* mutant strains. The *pezo-1 ( av240 )* allele is a full gene deletion, whereas *pezo-1 ( av149 )* and *pezo-1 ( sy1199 )* encode truncated proteins lacking transmembrane domains 29–38, which include the two transmembrane helices that form the channel pore ( Figure 1A ). We first assessed nose touch avoidance behavior in these mutants compared to wild-type (WT) animals. A modest reduction in response was observed in *pezo-1 ( av149 )* mutants (WT: 0.805 ± 0.02; av149 : 0.666 ± 0.046), whereas *pezo-1 ( av240 )* and *pezo-1 ( sy1199 )* mutants responded similarly to WT ( Figure 1B ). These results suggest that *pezo-1* does not play a major role in mediating nose touch avoidance behavior and that the small reduction in touch sensitivity seen in *pezo-1 ( av149 )* might be due to secondary mutations, given that this mutant has not been outcrossed.

Next, to determine whether *pezo-1* is required for mechanically evoked calcium transients in AMsh glia, we performed in vivo calcium imaging in *pezo-1 ( av240 )* animals expressing GCaMP-6s in AMsh glial cells ( Figure 1C ). In WT animals, nose touch stimulation elicited calcium transients in AMsh glia that exhibited adaptation upon a second stimulation, consistent with previous observations ( Figure 1D, E) (Chen et al., 2022; Duan et al., 2020; Fernandez-Abascal et al., 2022). Comparable responses were observed in *pezo-1 ( av240 )* mutants ( Figure 1G, H), with no significant difference in peak calcium signals and calcium kinetics between WT and mutant animals (ΔF/F, WT = 11.25 ± 2.87 and *av240* = 14.23 ± 5.45; tau, WT: 38.85 ± 5.71 and *av240* : 34.97 ± 4.65) ( Figure 1F, I). Together, these data indicate that *pezo-1* is not required for AMsh glial calcium responses to mechanical stimulation and does not play a central role in nose touch avoidance behavior. Future studies should focus on identifying alternative mechanosensitive channels or signaling pathways that contribute to mechanotransduction in AMsh glia.

## Methods

***C. elegans* growth and maintenance:** All *C. elegans* strains were maintained at 20°C on nematode growth medium (NGM) plates seeded with *Escherichia coli* OP50 , following standard protocols (Brenner, 1974). The Bristol N2 strain was used as the wild-type control. Only healthy, well-fed, day 1 (D1) young adult hermaphrodites were used in all experiments.

**Strains:** The following strains were used in this study: N2 , RB1052 *trpa-1 ( ok999 )* , AG416 *pezo-1 ( av149 )* , AG570 *pezo-1 ( av240 )* , PS811 *pezo-1 ( sy1199 )* , BLC402 *blcEx447[Pspig-1::GCaMP-6s;Punc-122::GFP]* , and BLC477 *pezo-1 ( av240 )* ; *blcEx447[Pspig-1::GCaMP-6s;Punc-122::GFP].* While strains AG416 *pezo-1 ( av149 )* and AG570 *pezo-1 ( av240 )* were generated by CRISPR/Cas9 and were not outcrossed, strain PS811 *pezo-1 ( sy1199 )* was outcrossed several times (Bai et al., 2020).

**Nose touch:** Behavioral assays were conducted as previously described (Fernandez-Abascal et al., 2022; Hart et al., 1999). Briefly, an eyelash tool was used to gently touch the nose of forward-moving animals, applied perpendicular to their direction of movement. Each worm received five consecutive touches, with a 30-second interval between stimuli. A reversal in response to the touch was scored as a positive response. All experiments were performed blind to genotype to avoid bias.

**Calcium imaging:** Nematodes expressing GCaMP-6s in AMsh glia were immobilized on 2% agarose pads made with extracellular saline solution, following previously established protocols (Fernandez-Abascal & Bianchi, 2022; Fernandez-Abascal et al., 2022; Johnson et al., 2020; Kindt et al., 2007). Imaging was performed using an Olympus IX70 microscope equipped with a Lambda DG-4 illumination system, an FF01-500/24-25 excitation filter (Semrock), and a Pco camera. Mechanical stimulation was applied to the tip of the nose using a borosilicate capillary glass probe, controlled by a C-863 Mercury Servo Controller (Physik Instrumente) and operated through PIMikroMover software (version 2.4.4.6). Each worm received two successive nose pokes. Imaging data were acquired using Micro-Manager 2.0 software (Edelstein, Tsuchida et al., 2014), with recordings starting 30 seconds before stimulation and lasting 135 seconds. Fluorescence data were analyzed using Fiji (ImageJ), and results were plotted using GraphPad Prism (version 8.4.2). Fluorescence signals were normalized to the average intensity recorded during the 10 seconds preceding stimulation.
